# Comment on “Ultrapotassic magmatism in the heyday of the Variscan Orogeny: the story of the Třebíč Pluton, the largest durbachitic body in the Bohemian Massif” by Janoušek et al.

**DOI:** 10.1007/s00531-020-01975-w

**Published:** 2021-02-13

**Authors:** U. Schaltegger, S. P. Gaynor, P. Widmann, J. Kotková

**Affiliations:** 1 Department of Earth Sciences, University of Geneva, rue des Maraîchers 13, 1205 Geneva, Switzerland; 2 Czech Geological Survey, Klárov, 118 21 Prague, Czech Republic; 3 Department of Geological Sciences, Masaryk University, Kotlářská 2, 611 07 Brno, Czech Republic

**Keywords:** Třebíč pluton, Moldanubian domain, Bohemian massif, Zircon, LA-ICP-MS, CA-ID-TIMS, U–Pb

## Abstract

This comment addresses the incorrect treatment and presentation of data from laser ablation ICP-MS U–Pb age determinations of two samples of the Třebíč Pluton. This results in inaccurate ages and error assessment, invalidating the age interpretations of the authors. To corroborate our arguments, new high-precision chemical abrasion ID-TIMS data are presented that unequivocally define the emplacement age of the Třebíč pluton.

The commented paper by Janoušek et al. (2020) presented an excellent and detailed assessment of the petrology, geochemistry and the mode of emplacement of the durbachitic Třebíč intrusion in the Bohemian Massif. The term “durbachite” denotes members of an intrusive series that comprises ultrapotassic, Mg-rich melagranites and ultrapotassic two-pyroxene melasyenites, which were emplaced into the exhumed high-grade core of the orogen during the late-orogenic stages (Žák et al. 2014). Among those is the Třebíč pluton in the Moldanubian domain of the Bohemian Massif, a tabular intrusion that has been emplaced at shallow crustal levels (corresponding to 2–4 kbar, Houzar and Novák 2006, Leichman et al. 2017), and whose flat-lying fabrics, which are transitional between magmatic and solid state, are concordant with the regional foliation in the host rocks (Janoušek et al. 2020). Therefore, its solidification dates the subvertical shortening of the Moldanubian domain, which is related to the underthrusting of the Brunia microcontinent (Verner et al. 2006; Žák et al. 2014). This is the reason why precise and accurate dating of the Třebíč pluton emplacement is important for the reconstruction of Variscan tectonics.

The dating of durbachite lithologies by zircon U–Pb has been described as problematic by many authors (e.g., Schaltegger et al. 1996; Klötzli and Parrish 1996), and this is re-stated by Janoušek et al. (2020) for the dating of the Třebíč pluton. We, however, feel that the dating of this intrusion is not as generally problematic as previously mentioned when appropriate techniques for isotope analysis and data processing are applied. We argue that: (1) inappropriate consideration of decay-damage related Pb-loss in non-chemically abraded zircon, coupled with inheritance of old Pb leads to the previously observed age variations and (2) inaccurate calculation and reporting of laser-ablation, inductively coupled plasma mass spectrometry (LA-ICP-MS) U–Pb ages and uncertainties in the recent paper of Janoušek et al. (2020) have contributed to an inaccurate and problematic geochronological data set. In this comment, we explain our concerns on these published data, re-interpret the dates, and present new, high-precision chemical-abrasion, isotope-dilution, thermal ionization mass spectrometry (CA-ID-TIMS) U–Pb dates that unequivocally constrain the age of zircon crystallization in the Třebíč melagranite melt.

Existing geochronology: we briefly discuss recent U–Pb geochronology from two papers, i.e., Kotková et al. (2010) and Janoušek et al. (2020).

*ID-TIMS dates in *Kotková et al. (2010)*:* These authors presented dates from three multigrain zircon fractions and 5 single zircon grains from the Třebíč pluton, classified in prismatic and tabular grains, all of which were mechanically abraded prior to dissolution and analysis. The prismatic grains were reported to host inherited, xenocrystic cores. While the tabular grains do not appear to have cores, they show clear signs of a secondary dissolution–reprecipitation processes (e.g., Geisler et al. 2007), indicative of potential post-crystallization Pb-loss. The presence of inheritance is highlighted by one grain with a ^207^Pb/^206^Pb date of 2.1 Ga, and a multigrain fraction yielding an age of 374 Ma. The interpreted zircon date relied on a Pb-loss line through four analyses, yielding an upper intercept age at 334.8 ± 3.2 Ma and a lower intercept indistinguishable from zero, documenting decay damage related Pb loss. It is a good example how problematic legacy data from mechanically abraded zircon multigrain fractions are, if both inheritance and Pb-loss were involved. The age calculation in Kotková et al. (2010) excluded an older multigrain fraction of 5 zircon crystals, based on the argument of unresolved inheritance within this single fraction. This interpretation is contested by Janoušek et al. (2020), arguing that the inclusion of the older fraction of prismatic grains yields an age interpretation of 338.5–339.5 Ma, which would coincide with their LA-ICP-MS dates at around 338–339 Ma.

*LA-ICP-MS dates in *Janoušek et al. (2020)*:* The authors have presented LA-ICP-MS dated of two samples of the main Třebíč intrusion, labelled 1322 and H003, with ages of 338.4 ± 2.8 and 335.0 ± 2.6 Ma, respectively. Unfortunately, the presentation of these data has a number of problems:

- The weighted mean ages of both samples are not concordant, and the calculated MSWD values of concordance are 23 and 11 for samples 1322 and H0003, respectively, using IsoplotR (Vermeesch 2018). Therefore, calculating a concordia age from these data is not appropriate. The reported MSWD values are, therefore, those of equivalence, rather than concordance. This elevated degree of sub-concordance may point to either (1) an analytical problem or (2) to unresolved common Pb contribution. The analytical procedures were previously described in Hanžl et al. (2019), mentioning “high Hg contamination of the commercially available He carrier gas” that made common Pb correction impossible. Therefore, we assume that the sub-concordance is an effect of ubiquitous, but unresolved, common Pb.
- Despite this, the authors report a “concordant age”, which is biased towards an artificially old ^207^Pb/^235^U age, due to the incorporation of common Pb. For such a case, reporting of the mean weighted ^206^Pb/^238^U age is more appropriate, combined with an MSWD value of equivalency. Computing weighted mean ^206^Pb/^238^U ages from the data set in Janoušek et al. (2020) yields values of 335.6 ± 2.5 Ma (MSWD = 1.52) for sample 1322, and of 334.2 ± 1.1 Ma (MSWD = 0.87) for sample H0003 (errors include overdispersion, but no constant external error from standard zircon measurement; Fig. 1a, b).
- In addition, it is evident that systematic external uncertainty, from the measurement of secondary standards, has not been propagated into the weighted mean ages. This is in disregard of the recommendations of Horstwood et al. (2016), a community manuscript published to maintain guidelines for the LA-ICP-MS analyses, such that all published data are accurate, comparable and valid. Hanžl et al. (2019) report an age deviation of their measured reference zircon materials Plešovice, GJ-1 and 91,500 of “less than 1.5%”; however, this deviation is not included in any error propagation. Adding to this argument, we introduce an additional estimated uncertainty of 2% for the above calculated mean ^206^Pb/^238^U dates, stemming from the age variation of the external reference material used to calibrate the U/Pb concentration ratio. A 2*σ* additional external uncertainty of 2% is in fact required to reach an MSWD of 1 in the case of sample 1322, while for sample H0003 an additional uncertainty of 1.3% is sufficient. Therefore, given these more reassuring statistics and unless proven otherwise, we base our further discussion on an uncertainty of approximately ± 6.7 Ma for each weighted mean age.

**Fig. 1:**
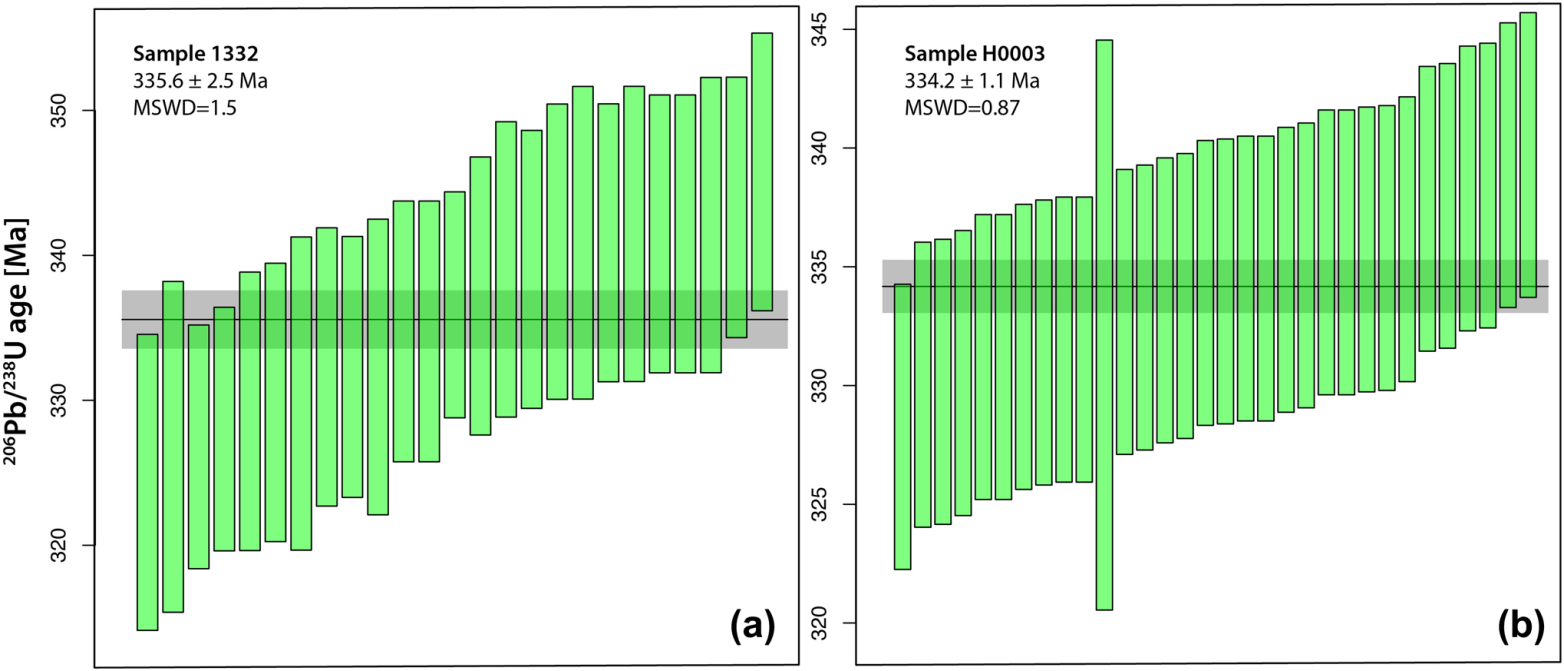
Ranked ^206^Pb/^238^U age plots for **a** sample 1322 and **b** sample H0003 from Janoušek et al. (2020)

Based on these new calculations, the two LA-ICP-MS dates from Janoušek et al. (2020) are at 335.6 ± 6.7 Ma and 334.2 ± 6.7 Ma, respectively, and are clearly indistinguishable from each other, as well as from the proposed upper intercept age of 334.8 ± 3.2 Ma in Kotková et al. (2010).

The temporal dispersion observed in all of the published zircon geochronology data sets from ultrapotassic rocks in the Moldanubian domain indicates that zircon grains are suffering from the following two effects:

(1) The radioactive decay damage-related loss of radiogenic Pb, due to elevated U concentrations (maximum 2050 ppm in Kotková et al. 2010; up to 3000 ppm from LA-ICP-MS analyses in Janoušek et al. 2020), leading to normal discordance and lowered ^206^Pb/^238^U dates. A high degree of lattice disturbance in decay-damaged zircon is visible through ubiquitous phenomena of dissolution–reprecipitation processes, leading to non-planar textures visible in CL (see Fig. 4i in Kotková et al. 2010, and our Fig. 2a) and radial cracks due to the volume increase during lattice disorder (Fig. 2b).

**Fig. 2:**
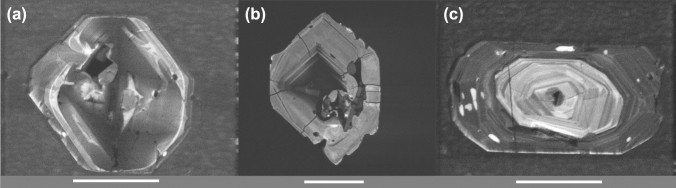
Representative cathodoluminescence images of zircon crystals from sample SU-05-2: **a** tabular crystal with non-planar zones pointing to secondary dissolution–reprecipitation processes; **b** tabular crystal with radial cracks; **c** prismatic zircon with low-luminescent, possibly older core. White length bars 50 µm

(2) The presence of inherited components as a consequence of crustal contamination, which has been previously identified by whole-rock trace elements and Sr, Nd, Pb isotopes in numerous Variscan durbachitic rocks (e.g., Tabaud et al. 2015) and by negative *εHf* values of − 4.3 to − 7.5 from zircon of the Třebíč intrusion (Kotková et al. 2010), leading to normal discordance and elevated ^206^Pb/^238^U and ^207^Pb/^235^U dates. The inheritance can be easily spotted by the presence of cores in the Třebíč sample SU-05-2 (see Fig. 4f in Kotková et al. 2010, and Fig. 2c).

The presence of these problems in durbachite zircon indicates that any technique that does not mitigate radioactive-decay damage related Pb-loss will be inaccurate, and may possibly mix both Pb-loss and inheritance into the same analysis. This is the case for any mechanically abraded ID-TIMS (as in Kotková et al. 2010), as well as for LA-ICP-MS and SIMS dates on untreated zircon.

Therefore, we carried out a series of new high-precision, CA-ID-TIMS analyses on sample SU-02-5 of the Třebíč intrusion, previously analyzed in Kotková et al. (2010), following the state-of-the-art U–Pb procedures described in detail in Widmann et al. (2019). Specifically, we utilized an optimized temperature of 210 °C for the partial dissolution step of the chemical abrasion procedure. While mitigation of Pb-loss has been demonstrated to peak with a 12-h pretreatment, the highly metamict nature of these grains causes them to undergo complete dissolution after 12 h, yielding no remaining zircon residue for chemistry and analysis. Therefore, we were forced to use a less robust 6-h partial dissolution, which allowed us to mitigate Pb-loss while still having remaining zircon material to date. The use of the ^202^Pb–^205^Pb–^233^U–^235^U Earthtime tracer solution helps to optimize both precision and accuracy for these analyses (Condon et al. 2015; McLean et al. 2015). The analyses were carried out on a Phoenix TIMS (IsotopX Ltd.) at University of Geneva, using Daly ion counting for dynamic measurement of Pb isotopes, and static measurement of UO_2_ on Faraday cups equipped by 10^12^ Ω resistance amplifiers.

Of the 14 zircons we dated by CA-ID-TIMS, 11 of the analyses are concordant and have Th-corrected ^206^Pb/^238^U ages ranging from approximately 335.8–338.0 Ma. Three analyses yielded normally discordant values at significantly older ages, with ^207^Pb/^206^Pb ages ranging up to 506 Ma. We interpret the presence of these older dates as indicative of xenocrystic components present within these zircons from the Třebíč pluton, and subtle incorporation of these domains artificially drives zircon ages to values older than the crystallization of the pluton. Based upon this, we interpret that the older cluster of overlapping ages (Z12, Z11, Z6) to also incorporate inheritance, albeit to a lesser degree. One single analysis is younger than the rest, which we interpret to reflect incomplete mitigation of Pb-loss due to the shortened abrasion.

Therefore, we interpret that the six overlapping analyses at approximately 335 Ma reflect the crystallization of the Třebíč Pluton, with the other analyzed crystals reflecting the aforementioned problems. From those 6 grains, we obtained a weighted mean ^206^Pb/^238^U age of 335.127 ± 0.061 Ma (± 0.11 Ma, including propagated systematic uncertainties; MSWD = 1.7, Table 1, Fig. 3).

**Table 1:** U–Pb CA-ID-TIMS age determinations from zircon of the Třebíč pluton

Fraction	Composition					Isotopic ratios							Dates (Ma)							
	U (ng)	Th/U^a^	Pb* (pg)^b^	Pb^c^ (pg)^c^	Pb*/Pb_c_^d^	^206^Pb/^204^Pb^e^	^206^Pb/^238^U^f^	± 2σ %	^207^Pb/^235^U^f^	± 2σ %	^207^Pb/^206^Pb^f^	± 2σ %	^206^Pb/^238^U^g,h^	± 2σ abs	^207^Pb/^235^U^h^	± 2σ abs	^207^Pb/^206^Pb^h^	± 2σ abs	Corr. coef.	% Disc^i^
SU-02-05_2	0.99	0.26	51.49	0.96	53.86	3328	0.053334	0.053	0.389790	0.166	0.053030	0.152	335.05	0.17	334.22	0.47	329.06	3.53	0.35	− 1.79
SU-02-05_3	2.15	0.25	111.80	0.63	177.73	10,964	0.053328	0.037	0.390412	0.072	0.053121	0.051	335.02	0.12	334.67	0.21	332.94	1.37	0.55	− 0.59
SU-02-05_4	0.37	0.13	20.40	0.50	41.11	2636	0.059044	0.044	0.442874	0.197	0.054425	0.190	369.91	0.16	372.28	0.61	387.67	4.32	0.21	4.61
SU-02-05_5	3.44	0.27	180.11	0.93	193.16	11,829	0.053350	0.040	0.390935	0.073	0.053170	0.049	335.15	0.13	335.05	0.21	335.02	1.33	0.60	− 0.01
SU-02-05_6	0.88	0.28	46.35	0.58	80.39	4923	0.053502	0.040	0.392601	0.109	0.053244	0.101	336.08	0.13	336.27	0.31	338.19	2.41	0.26	0.65
SU-02-05_7	0.36	0.32	19.24	1.05	18.35	1126	0.053346	0.070	0.392094	0.401	0.053331	0.395	335.13	0.23	335.90	1.15	341.90	8.97	0.16	2.01
SU-02-05_8	0.24	0.27	15.39	3.25	4.74	306	0.064046	0.154	0.502502	1.317	0.056930	1.319	400.28	0.60	413.39	4.47	487.83	29.11	0.04	17.97
SU-02-05_9	0.80	0.33	42.44	1.53	27.77	1687	0.053368	0.041	0.392276	0.255	0.053334	0.250	335.26	0.13	336.03	0.73	342.02	5.71	0.14	2.00
SU-02-05_10	0.36	0.25	18.81	1.10	17.08	1068	0.053770	0.157	0.395671	0.441	0.053393	0.412	337.72	0.52	338.50	1.27	344.52	9.34	0.35	2.00
SU-02-05_11	0.17	0.15	8.37	0.58	14.39	929	0.053465	0.114	0.389600	0.545	0.052875	0.527	335.86	0.37	334.08	1.55	322.40	12.00	0.25	− 4.14
SU-02-05_12	0.16	0.49	8.71	0.90	9.70	577	0.053462	0.091	0.391239	0.794	0.053099	0.787	335.83	0.30	335.27	2.27	332.02	17.86	0.12	− 1.12
SU-02-05_13	1.07	0.27	55.61	0.41	136.72	8389	0.053353	0.058	0.391096	0.098	0.053188	0.069	335.17	0.19	335.17	0.28	335.82	1.73	0.63	0.22
SU-02-05_14	0.10	0.56	7.88	0.50	15.74	907	0.077302	0.061	0.611427	0.462	0.057392	0.462	480.08	0.28	484.46	1.78	505.63	10.20	0.04	5.07
SU-02-05_15	0.78	0.26	40.71	0.93	43.90	2713	0.053266	0.106	0.390233	0.209	0.053158	0.172	334.64	0.35	334.54	0.60	334.52	3.97	0.54	− 0.01

^a^Th contents calculated from radiogenic ^208^Pb and ^230^Th-corrected ^206^Pb/^238^U date of the sample, assuming concordance between U–Pb Th–Pb systems

^b^Total mass of radiogenic Pb

^c^Total mass of common Pb

^d^Ratio of radiogenic Pb (including ^208^Pb) to common Pb

^e^Measured ratio corrected for fractionation and spike contribution only

^f^Measured ratios corrected for fractionation, tracer, blank and, where applicable, initial common Pb

^g^Corrected for initial Th/U disequilibrium using radiogenic ^208^Pb and Th/U_[magma]_ = 3.50

^h^Isotopic dates calculated using *λ*_238_ = 1.55125E−10 (Jaffey et al. 1971) and *λ*_235_ = 9.8485E−10 (Jaffey et al. 1971)

^i^% discordance = 100 − (100 × (^206^Pb/^238^U date)/(^207^Pb/^206^Pb date))

**Fig. 3:**
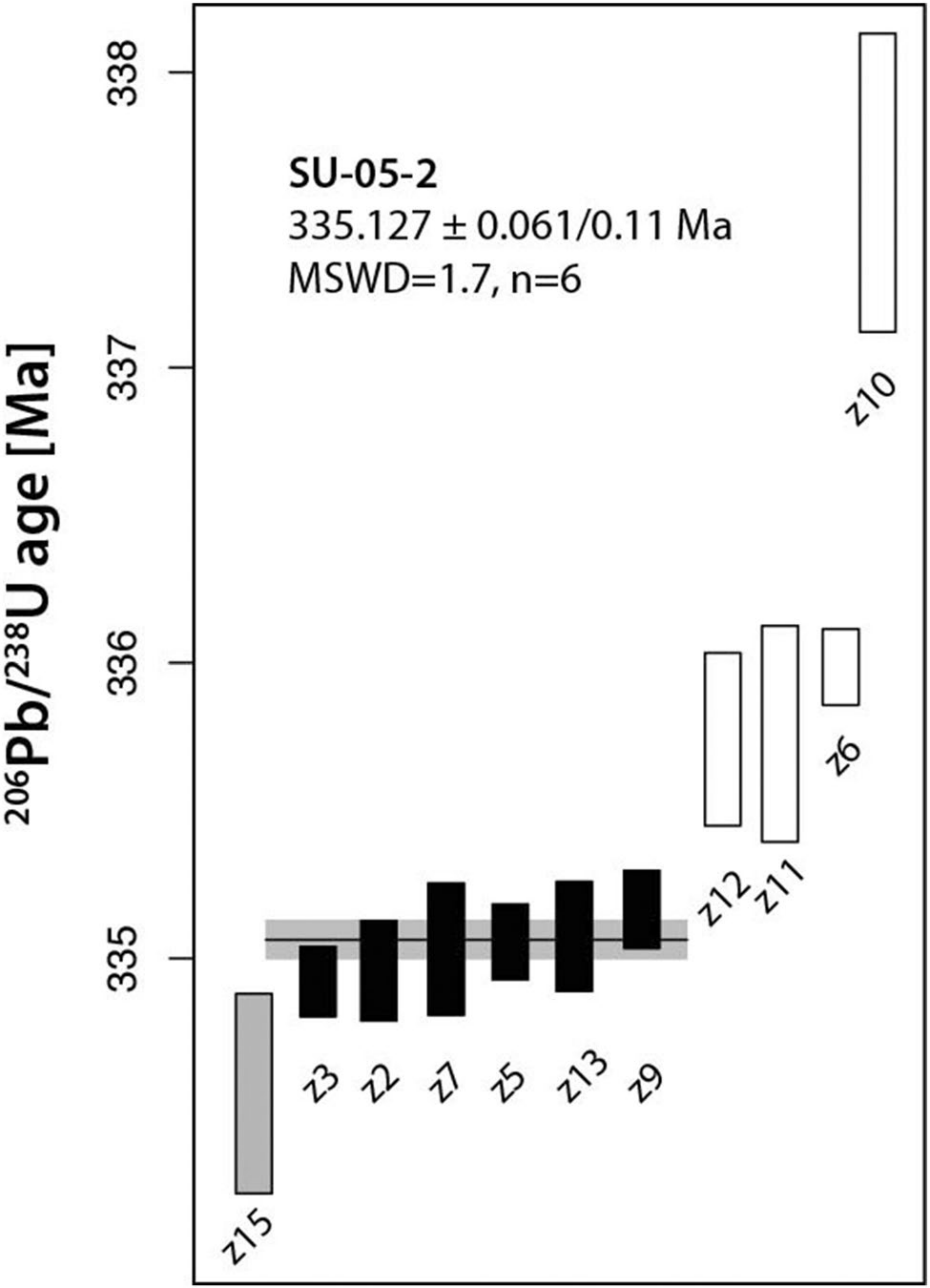
Ranked ^206^Pb/^238^U age plot with CA-ID-TIMS dates from sample SU-02-5. Analyses with black error bars are considered for calculating the weighted mean age. Analyses z4, z8 and z14 (Table 1) yield discordant ages > 339 Ma indicative of the presence xenocrystic material and are not shown. The zircon plotted in gray is interpreted to reflect some amount of unmitigated Pb-loss, while the analyses shown in white are interpreted to incorporate trace xenocrystic material, and therefore, those five analyses are not included in the weighted mean calculation

From the above arguments, we conclude the following:

(1) All ^206^Pb/^238^U dates from mechanical-abrasion ID-TIMS and LA-ICP-MS geochronology perfectly overlap at around 335 Ma when uncertainties are correctly considered. This age is duplicated by a new CA-ID-TIMS at 335.127 ± 0.061/0.11 Ma (internal/external uncertainty).

(2) The zircon crystals contain components of radiation damage-related Pb-loss, which can be mitigated by thorough chemical abrasion; however, the reported LA-ICP-MS dates were measured on untreated samples. The LA-ICP-MS community should eventually proceed to analysis of chemically abraded sample and standard material, given the increased accuracy provided by the technique (e.g., Crowley et al. 2014; von Quadt et al. 2014; Watts et al. 2016).

(3) The intrusion of the Třebíč Pluton is confirmed to have taken place at 335.1 Ma, which at the same time constrains the formation age of the flat-lying fabrics. We cannot exclude the possibility that the satellite Drahonín intrusion is older than the main intrusive volume, as it is reported in Janoušek et al. (2020); however, this will require additional geochronology.

## Data Availability

The raw data from mass spectrometry can be obtained on request from the lead author.
